# Case Report: Off Label Utilization of Topiramate and Metformin in Patients With BMI ≥50 kg/m^2^ Prior to Bariatric Surgery

**DOI:** 10.3389/fendo.2021.588016

**Published:** 2021-02-25

**Authors:** Cetin Sari, Richard L. Seip, Devika Umashanker

**Affiliations:** ^1^ Metabolic and Bariatric Surgery Center, Hartford Hospital, Hartford, CT, United States; ^2^ Division of Research Data Management, Hartford Hospital, Hartford, CT, United States; ^3^ Medical Weight Management Program, Hartford Hospital, Hartford, CT, United States

**Keywords:** generic weight loss medications, preoperative weight loss, anti-obesity pharmacotherapy, pre-surgery, super obesity, medical weight loss, MBSAQIP surgical risk calculator

## Abstract

FDA approved anti-obesity medications may not be cost effective for patients struggling with pre-operative weight loss prior to bariatric surgery. Metformin, a biguanide, and Topiramate, a carbonic anhydrase inhibitor, both cost effective medications, have demonstrated weight loss when used for the treatment of type 2 diabetes or seizures, respectively. The aim of the three cases is to demonstrate the clinical utility of topiramate and metformin for preoperative weight loss in patients with a body mass index (BMI) ≥ 50 kg/m^2^ prior to bariatric surgery who are unable to follow the bariatric nutritional prescription due to a dysregulated appetite system Each patient was prescribed metformin and/or topiramate in an off-label manner in conjunction with lifestyle modifications and achieved >8% total body weight loss during the preoperative period.

## Introduction

Bariatric surgery is considered a safe and effective treatment for most patients with obesity. For patients with severe obesity, whose body mass index (BMI) exceeds 50 kg/m^2^, the benefits of bariatric surgery must be weighed against perioperative complication risks which are known to be higher independent of surgical technique (1–3). The primary strategy for minimizing complications in such patients is to decrease the BMI before surgery. Preoperative weight loss through dietary modification has demonstrated reduced surgical morbidity for patients with severe obesity (4). Pharmacotherapy using FDA-approved anti-obesity medications (AOM) such as Phentermine/Topiramate (Qsymia^®^), Bupropion/Naltrexone (Contrave^®^), Liraglutide 3.0 (Saxenda^®^), or Orlistat (Xenical^®^) may yield 5%–10% total body weight loss (%TWL) (5), exceeding the 3% seen with behavioral modification alone (6). Since FDA approved anti-obesity medications (AOMs) may be costly and usually are not covered by insurance, lower cost medications such as metformin and topiramate, which have the side effect of weight loss in many patients, warrant consideration as medical therapy for preoperative weight loss patients with BMI ≥ 50 kg/m^2^.

Here, we describe three cases that illustrate short term weight loss results following treatment using the selective off-label use of topiramate and metformin, under the guidance of an obesity medicine specialist (OMS).

## Material and Methods

Patients with a BMI ≥ 50 kg/m^2^ who were candidates for bariatric surgery were referred to see an OMS. Each signed a consent permitting the use of his or her medical data. Each patient met the criteria for initiation of AOM which is BMI >27 kg/m^2^ plus the presence of one obesity-related comorbidity and/or BMI >30 kg/m^2^. The AOM were prescribed in conjunction with lifestyle intervention, as part of the bariatric surgery pre-operative assessment. A bariatric program registered dietician recommended dietary macronutrient intake in the range of 40% carbohydrates, 30% total fat of which <10% should be polyunsaturated fats, and 30% protein (7). In addition, each patient was advised to engage in at least 150 min per week of structured moderately intensive exercise per the American College of Sports Medicine guidelines (8), or if unable, referred to physical therapy.

After a complete medical and psychiatric history, patients were treated either with topiramate or metformin or both in combination since these patients did not have insurance coverage nor could afford the FDA approved weight loss medications. For each case, body weights recorded at clinic visits were plotted versus time, with medication changes noted on the timeline.

### Medication Cost Tabulation

The prices for a single month supply of weight loss medications with and without coupons were acquired through online query of GoodRx^®^, a website which compiles medication costs at various pharmacies throughout the United States (9). GoodRx^®^ tabulates average cash price for prescription drugs from different pharmacies, presenting a 5-month history of price with and without coupons. Data presented in **Table 1** were obtained using this feature of the GoodRx^®^ website. This website is frequently utilized by our practice to help patients find the most affordable prices for their medications.

**Table 1:** Prescription drug costs of FDA approved anti-obesity medications vs topiramate and metformin.

Medication	Estimated Cost	
	Average Monthly	With GoodRx^®^ Coupon
Saxenda^®^ (Liraglutide 3.0)	$1,531.58	$1,287.90–$1,364.21
Qsymia^®^ (Phentermine/Topiramate)	$231.64	$191.87- $208.56
Contrave^®^ (Buporopion/Naltrexone)	$334.45	$272.10
Xenical^®^ (Orlistat)	$818.32	$611.86–$742.11
topiramate	$45.48	$6.11–$53.45
metformin	$16.22	$3.13–$15.44
phentermine	$35.98	$17.24

Cost data were obtained from Goodrx.com as of April 2020 (9).

## Case Scenarios

### Patient A

A 32-year-old female with past medical history (PMH) of Class IV obesity, metabolic syndrome, anxiety, and depression was referred to the medical weight management program for weight loss prior to laparoscopic sleeve gastrectomy (LSG). BMI was 51.2 kg/m^2^. The patient was prescribed the bariatric nutritional plan per surgical team and recommended to follow the American Sport Medicine recommendations of 150 min of moderate exercise per week. Prior to OMS consult, glucose was elevated at 100 mg/dl and HDL cholesterol at 45 mg/dl was low. Tests of liver function were normal, as were other measures within the panel. During OMS consult, the patient reported difficulty following the nutritional plan and was unmotivated to exercise. Her nutritional challenges were carbohydrate cravings resulting in overeating. The patient’s medication list was reviewed, revealing no obesogenic medications contributing to weight gain. She reported having taken metformin and topiramate several years ago, metformin for treatment of possible history of polycystic ovarian syndrome (PCOS) and to assist with weight loss (which the patient was unable to confirm), and topiramate for treatment of anxiety and depression. She self-discontinued both due to intolerable side effects. Metformin 500 mg BID caused diarrhea and topiramate 200 mg daily caused cognitive dullness.

**Figure 1:**
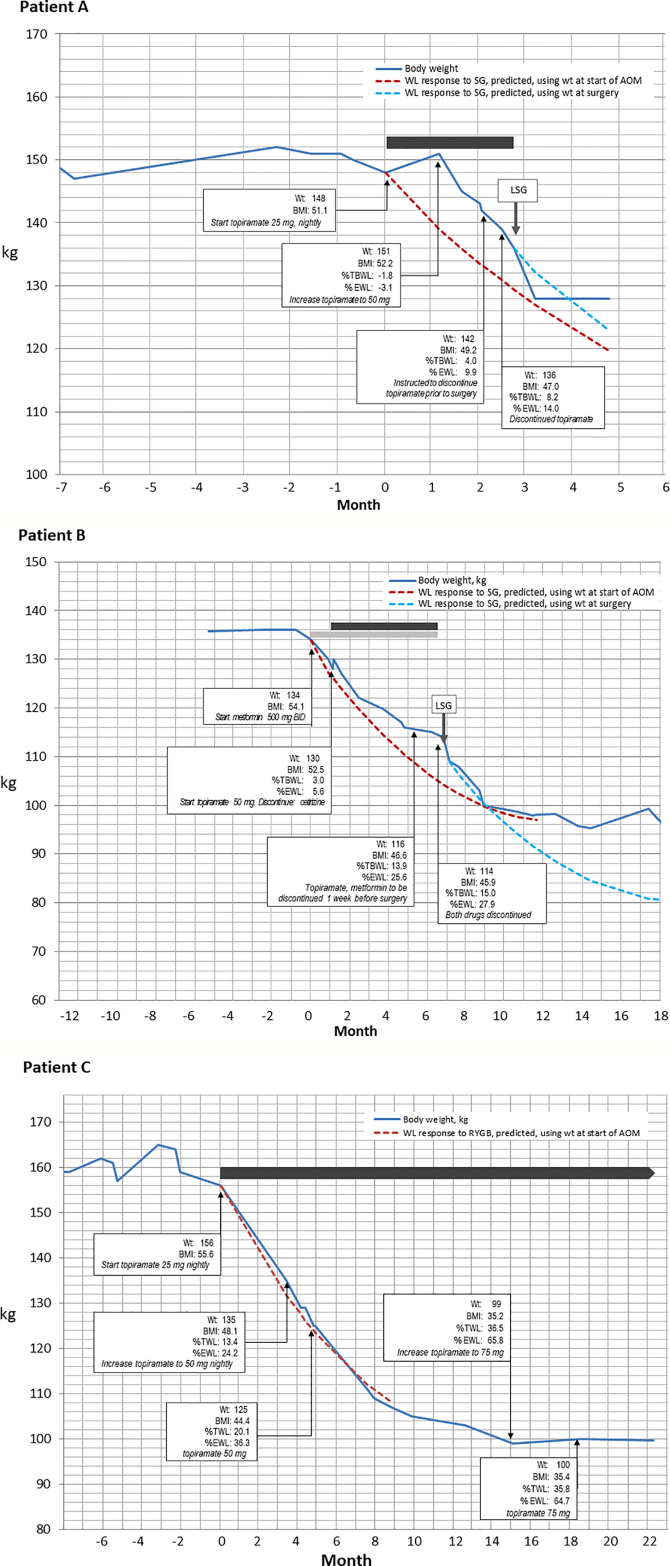
Changes in body weight over time (blue line) for three patients who received medical weight loss pharmacotherapy prior to bariatric surgery. In all three panels, dashed lines depict the weight loss that is predicted by the MBSAQIP risk calculator accessible at https://riskcalculator.facs.org/bariatric/patientoutcomes.jsp (10). **(A)** A 12 kg weight loss in response to 12 weeks of topiramate therapy in a 32-year-old woman. **(B)** A weight loss of 18 kg over 6 months of combination therapy with metformin and topiramate, prior to laparoscopic sleeve gastrectomy in a 58-year-old woman. **(C)** Careful alteration of topiramate dosing to induce a 56 kg weight loss in a 39-year-old woman, which resulted in the patient electing to forego surgery.

At the initial medical weight management visit with the OMS, she was started on low dose topiramate 25 mg daily with a goal to decrease carbohydrate cravings while avoiding previously experienced side effects. Exercise at 10 min of walking per day was prescribed. She was not started on Metformin due to side effect intolerance and patient refusal. One month later she had gained 2.72 kg due to persistent cravings without abatement of hunger drive even though she had increased physical activity and tried to follow the bariatric nutrition plan. As a result, topiramate was increased to 50 mg per day. At Visit #3 with the OMS 10 weeks later, the patient lost 8.62 kg and reported a reduction in carbohydrate craving and decreased hunger drive. She denied side effects related to topiramate such as paraesthesias, dizziness, dysgeusia, insomnia, constipation, and dry mouth. Total weight loss in response to 12 weeks of topiramate therapy prior to bariatric surgery was 14.97 kg, [8.2% total body weight loss (%TBWL)]. The patient advanced to LSG at BMI 47.0 kg/m^2^. A metabolic panel obtained on day of surgery revealed fasting glucose of 101mg/dl, minimal difference from initial consult lab values. A lipid panel was not obtained. Post-surgery 3-month lab results were pending at this writing. The patient’s surgical course was without intraoperative complications and her length of hospital stay was one overnight without post-operative complications. Patient anti-hypertensive medication initiated during the bariatric surgical work up was discontinued after surgery due to well controlled blood pressure readings. This patient’s weight loss progression appears in **Figure 1A**.

### Patient B

A 58-year-old female with PMH of Class IV obesity, metabolic syndrome, prediabetes, obstructive sleep apnea (OSA) adherent to CPAP, and depression was referred to the medical weight management program for weight loss prior to LSG. BMI was 52.2 kg/m^2^. The patient was prescribed the bariatric nutritional plan per surgical team and advised to follow the American College of Sports Medicine recommendations of 150 min of moderate exercise per week. Blood lipid panel prior to medical weight loss management showed triglycerides 167 mg/dL, HDL 48 mg/dl, HbA1c 6.1%, and fasting glucose 116 mg/dL. The patient was unable to follow the nutritional plan due to a strong hunger drive with late-night eating after 10 pm. She reported following a low impact cardiovascular fitness plan (30 min, 5x/week) and practicing yoga. Medications at this time included two obesogenic medications, fluoxetine for the treatment of depression and cetirizine for seasonal allergies. Fluoxetine controlled her mood, and thus was continued, but cetirizine was discontinued and replaced with a prescription for a weight neutral antihistamine, loratadine. She was naïve to weight loss medications prior to starting the surgical weight loss program. At the initial bariatric consult, the bariatric surgeon prescribed metformin 500 mg twice daily for appetite reduction and at the medical weight management consult the OMS prescribed topiramate 50 mg nightly to reduce late night eating. This combination therapy, per patient report, reduced appetite and late-night eating. She denied side effects related to metformin (i.e., constipation, diarrhea, or abdominal bloating) and topiramate (somnolence, cognitive dullness, paresthesia, or dysguesia). After a total of 3 visits with the OMS, and prescription of metformin and topiramate for 6 months, the patient’s total weight loss prior to surgery was 20.41 kg, 15.0% TBWL, resulting in a BMI of 45.9 kg/m^2^ at the time of LSG surgery. No intraoperative or postoperative complications occurred, and length of hospital stay was one overnight. **Figure 1B** depicts the weight loss timeline for patient B. After medical and surgical weight loss, HbA1c was 5.4%, and fasting glucose, triglycerides and HDL-cholesterol were 70, 90, and 63 mg/dL, respectively.

### Patient C

A 39-year-old female with PMH of Class IV obesity and vitamin B_12_ deficiency was referred to the medical weight management program prior to LSG. The patient experienced a 9 kg weight gain during the bariatric surgical work up, being unable to adhere to the bariatric nutrition plan or physical activity plan prescribed by the surgical team. BMI was 55.5 kg/m^2^. Blood chemistry review at the time of initial bariatric consult showed triglycerides 186 mg/dl and HDL cholesterol 42 mg/dl which six months later improved to 106 mg/dl and 49 mg/dl, respectively. Her nutritional challenges were increased appetite, carbohydrate cravings, and depressive symptoms related to her weight which resulted in overeating. She was unable to follow the physical activity plan due to time restrictions. Review of the patient’s medication list was revealed no obesogenic medications contributing to weight gain. A review of her medical record showed she was weight loss medication naïve. She was therefore started on topiramate 25 mg to reduce overall hunger drive. Topiramate 25 mg increased satiety, reduced cravings, and indirectly improved her mood. After five months, the topiramate dose was adjusted to 50 mg to combat an increase in her hunger drive. She had lost a total of 31.30 kg, 20.1% TBWL, leading to BMI of 44 kg/m^2^. Topiramate was adjusted to 75 mg to address increase in hunger drive at month 15. After 22 months on topiramate, total weight loss was 56.70 kg (36% TBWL), resulting in a BMI of 35.4 kg/m^2^. While on topiramate, patient denied side effects of cognitive dullness, paresthesia, dysgeusia.

As a result of this robust weight loss, her surgical work up was placed on hold. Patient C’s weight loss is graphed in **Figure 1C**. Of note, the patient actual weight loss with topiramate was similar to the projected weight loss of RYGB as predicted by the MBSAQIP risk calculator (11).

## Discussion

Severe obesity has higher surgical morbidity and results tend to be worse compared to Class 3 obesity independent of surgical technique. The best approach to treat severe obesity has not been established. Although bariatric surgery is the gold standard treatment for obesity, our case series explores the potential role of affordable generic medications such as topiramate and metformin in selected patients with Class IV obesity and suggests, at least in these patients, the approach can be a safe strategy to facilitate weight loss prior to surgery.

The approach for utilizing medications to augment pre-operative weight loss is a multi-step process starting with the review of the patient’s past medical history and identification & adjustment of obesogenic medications. Next, anti-obesity medications are selected based on factors judged by the treating clinician to be barriers preventing adherence to the prescribed diet. In our series, metformin and topiramate were selected to modify hunger drive, including reduction of cravings and increasing feelings of satiety. The drugs can be used alone or in combination to facilitate pre-operative weight loss. The mechanisms of action through which the drugs reduce dietary energy intake differ, and the clinician may consider them as complementary tools in the management of weight loss.

Metformin’s effect to decrease dietary energy intake may involve more than one mechanism. Metformin has an AMP kinase-dependent effect on glucagon-like peptide-1 (GLP-1)–secreting L cells and increases postprandial GLP-1 secretion, which seems to contribute to its glucose-lowering effect and weight loss effects (12). Also, studies in rodents have shown increasing expression and secretion of growth differentiating factor 15 (GDF15) in hepatocytes leading to decreased appetite and subsequent weight loss (13). Topiramate, which is clinically prescribed for migraine prophylaxis and treatment of seizures, induces weight loss *via* appetite suppression. Appetite suppression is secondary to modulation of voltage-gated ion channels, increased activity of γ-aminobutyric acid (GABA)-A receptor, and inhibition of α-amino-3-hydroxy-5-methyl-4-isoxazolepropionic acid (AMPA)/kainite glutamate receptors (6, 14).

All patients achieved at least 8% TBWL with topiramate and/or metformin prior to intended bariatric surgery. Regardless of preoperative weight loss continuing to be a debated topic due to contradictory publications, clinically there are multiple advantages of implementing pre-operative weight loss in patients with obesity. Preoperative weight loss has demonstrated decrease in the size of the liver and the thickness of the abdominal wall, allowing the surgery to be technically easier (15). Excess visceral fat and a high liver volume are known to complicate the technical aspects of bariatric surgery because they can increase the blood loss volume, operating time, and risk of complications (16, 17). There may also be significant 30-day mortality risk reduction postoperatively associated with moderate weight loss of <5% (18). Pre-operative weight loss in patients with obesity may reduce the increased likelihood of hypoxemic complication due to reduction in apneic oxygenation reserve (19). Preoperative weight loss in patients with severe obesity allows patients to become safer candidates for bariatric surgery which may have not been the case without this presurgical intervention allowing patients to proceed with bariatric surgery and changing their quality of life.

Some studies have questioned the use of pre-operative weight loss as it does not necessarily correlate with postoperative weight loss (20, 21). One argument against the strategy of medical weight loss prior to surgery is that such weight loss may serve to reduce the amount of weight loss due to surgery. However, both medical and surgical therapies contribute to total weight lost and it is reasonable that applying medical therapy in tandem with surgery should increase the likelihood of maximizing the individual’s weight loss outcome and hence reducing weight related co-morbidities and improving overall health (6, 17).

## Conclusion

Reducing body weight in patients with severe obesity to decrease the risks of bariatric surgery is challenging. Treatment involving nutritional and behavioral change, alone or in combination prior to surgery, may fail to provide the desired preoperative weight loss. The findings of this small cases series seem to indicate that use of topiramate and metformin can achieve clinically significant weight loss prior to surgery in patients with a BMI ≥ 50 kg/m^2^.

## Data Availability Statement

The raw data supporting the conclusions of this article will be made available by the authors, without undue reservation.

## Ethics Statement

Written informed consent was obtained from the participants for the publication of any identifiable data or images in the article.

## Author Contributions

CS summarized the case histories and was responsible for the evolution of the manuscript. RS supervised data collection, conducted statistical analyses, constructed figures, and contributed to writing the manuscript. DU managed and treated 100% of the cases described in the manuscript, originated the concept for the manuscript, contributed to the discussion, and oversaw overall development of the manuscript. All authors contributed to the article and approved the submitted version.

## Conflict of Interest

The authors declare that the research was conducted in the absence of any commercial or financial relationships that could be construed as a potential conflict of interest.
